# Study of Different Sol-Gel Coatings to Enhance the Lifetime of PDMS Devices: Evaluation of Their Biocompatibility

**DOI:** 10.3390/ma9090728

**Published:** 2016-08-25

**Authors:** María Aymerich, Ana I. Gómez-Varela, Ezequiel Álvarez, María T. Flores-Arias

**Affiliations:** 1 Photonics4 Life Research Group, Departamento de Física Aplicada, Facultad de Física, Universidade de Santiago de Compostela, Santiago de Compostela 15782, Spain; maria.aymerich@usc.es (M.A.); anaisabel.gomez@usc.es (A.I.G.-V.); 2 Instituto de Investigación Sanitaria de Santiago de Compostela (IDIS), Complexo Hospitalario Universitario de Santiago de Compostela (CHUS) SERGAS, Santiago de Compostela 15706, Spain; ezequiel.alvarez.castro@gmail.com

**Keywords:** sol-gel, laser writing technique, soft lithography, PDMS devices, cell adhesion

## Abstract

A study of PDMS (polydimethylsiloxane) sol-gel–coated channels fabricated using soft lithography and a laser direct writing technique is presented. PDMS is a biocompatible material that presents a high versatility to reproduce several structures. It is widely employed in the fabrication of preclinical devices due to its advantages but it presents a rapid chemical deterioration to organic solvents. The use of sol-gel layers to cover the PDMS overcomes this problem since it provides the robustness of glass for the structures made with PDMS, decreasing its deterioration and changing the biocompatibility of the surface. In this work, PDMS channels are coated with three different kinds of sol-gel compositions (60MTES/40TEOS, 70MTES/30TISP and 80MTES/20TISP). The endothelial cell adhesion to the different coated devices is evaluated in order to determine the most suitable sol-gel preparation conditions to enhance cellular adhesion.

## 1. Introduction

The fabrication of preclinical devices, such as microfluidic or lab-on-a-chip systems, has had a huge impact and has received great interest due to the wide variety of medical and biological applications that these devices present. From diagnosis [1] to the study of flow conditions with different cardiovascular systems [2], these kinds of devices have a huge potential to carry out the simulation of pathologies in a small area and under controlled conditions [3]. These artifacts can mimic biological structures such as small capillaries [4], larger vessels [5,6], a simple channel or a complex miniaturized laboratory [7,8].

For these systems, a wide range of materials are employed, including glass [9], silicon [10] or thermoplastic polymers [11], among others. One of the most suitable materials for the fabrication of the chip devices is polydimethylsiloxane (PDMS) [12] due to its capability to replicate structures with accuracy down to the nanometer scale. It is a cheap material with a good permeability for gases which allows cells to receive oxygen when they are cultured on PDMS chips. Its optical transparency allows the necessary microscopy inspections. Its composition does not present any toxicity and it is biocompatible.

Different techniques have been reported for fabricating these kinds of devices, for instance photolithography [13] or laser micromachining [14]. When using PDMS, the most common and implemented replication technique is soft lithography [15,16], which allows us to obtain an accurate replica of a determined master in an easy and fast way. Masters can also be obtained by other methods, such as cutting plotter [17] or lithography [18]. Among all the possible techniques, laser structuring stands out due to its accuracy, speed, versatility and non-contact nature [19]. Laser ablation is commonly carried out with nanosecond pulsed lasers, which are the more implemented in the industry than those that operate in the femtosecond regime. Some of the authors have studied the laser direct writing technique over glass, which presents itself as a very suitable material for master fabrication mainly due to its hardness and resistance [20].

As we mentioned before, microfluidic and preclinical devices are commonly fabricated with PDMS due to its advantages but it also presents one important disadvantage: the degradation of the material when organic solvents are used. These substances, which are commonly employed in biomedical assays, degrade and deteriorate the PDMS, making the device non-reusable. A solution to overcome this problem is to coat the PDMS chips via the sol-gel chemical route. This procedure provides the structure with the chemical robustness of the glass and preserves the biocompatibility and transparency properties without significantly altering the geometry of the PDMS device [21,22]. Sol-gel chemistry offers some unique opportunities for the synthesis of optical materials over existing production methods including the control of the composition and the low processing temperature [23,24,25]. The process gives excellent control of its purity and composition since it starts with pure materials.

The dip-coating technique is one of the easiest techniques to obtain coatings using a liquid deposition with a huge variety of inorganic, hybrid and nanocomposite materials. It allows us to deposit layers and to coat different complex surfaces, for instance with holes or intricate shapes, enabling a flexibility that is not possible with other conventional techniques. By coating PDMS devices with sol-gel, the geometry of the devices can be preserved but the surface where cells are going to be cultured is modified and, therefore, cellular behavior over the material will be different compared to the behavior over non-coated PDMS. The composition of the cover layers can be chosen to enhance the cell adhesion.

In this work we present the fabrication of PDMS devices with channel geometry that can mimic blood vessels and may be employed in biomedical assays. The proposed chips are fabricated in a process of several steps, explained in detail in the Results section. The master is fabricated with laser technologies and it is replicated by soft lithography methods. After that, the PDMS devices are coated with different sol-gel compositions to overcome the deterioration problem. Human umbilical vein endothelial cells (HUVEC) have been chosen to be cultured onto the different coated channels. The biocompatibility of each specific device is studied. A comparison among them is presented when endothelial cells are seeded over the chips. Section 2 is devoted to the results obtained in the fabrication procedure and the biological validation. Section 3 shows the discussion of the results and the main conclusions of the work. Section 4 describes the materials and methods employed. 

## 2. Results

In this section, a technique that combines laser and soft lithography to fabricate the master and the replica of the channels, respectively, is presented. The channels are coated with different compositions of sol-gel. The biocompatibility of each coating is evaluated in order to find the most suitable sol-gel coating that reduces the deterioration of the PDMS [26,27] as well as to present the best environment for endothelial cells to attach and spread. These are key factors to perform successful bioassays with PDMS devices coated with sol-gel.

### 2.1. Fabrication of the Channel

Two steps compose the fabrication procedure of the final channels: first, a master of the channel is fabricated over a soda-lime glass with a pulsed nanosecond laser; second, a laser thermal treatment is applied to reduce the roughness of the channels. Once the master is fabricated, the replica procedure is carried out by soft lithography methods using PDMS material because of its biocompatibility.

#### 2.1.1. Master Fabrication with Laser Technologies

Due to its versatility for creating different geometries and to its accuracy, laser ablation is a very well-suited technique for fabricating a channel over soda-lime glass that will act as a master sample. To perform the structuring of the glass, a Nd:YVO_4_ laser with a fundamental wavelength of 1064 nm and pulses of 20 ns in the Q-switch regime is employed. The laser setup is combined with a galvanometer system that addresses the output beam. In order to focus the laser beam, a flat field lens with a 100 mm focal length is employed. This lens provides a working area of 80 × 80 mm^2^. Laser optimal parameters for the fabrication of the structure are: average power of 8 W, repetition rate of 12 kHz and scan speed 1000 mm/s.

**Figure 1:**
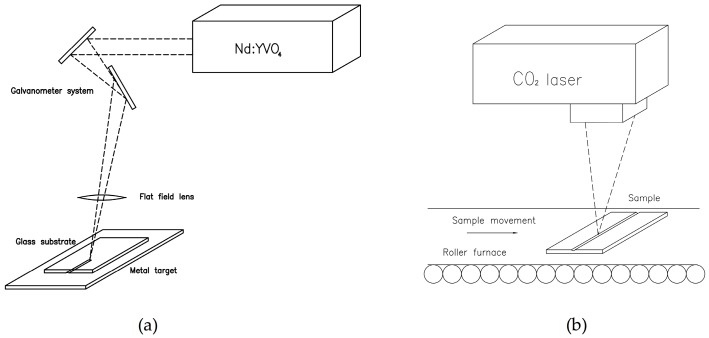
Experimental setups employed for the fabrication of the channel over soda-lime glass. Schemes of (**a**) the fabrication of the channel; and (**b**) the subsequent thermal treatment to reduce the roughness of the structure.

To achieve a channel with a depth on the order of millimeters, a laser backwriting process is applied in the manufacturing process [28]. A metal foil is placed below the glass substrate and the laser is focused in the metal target. The ablation is produced in the lower face of the soda-lime glass by the ablation plume, which expands from the metal to the glass. This technique allows us to create structures from microns to millimeters. In order to reduce the roughness of the channel occurring in this ablative process and to enhance the optical quality of the final device, a thermal treatment using a CO_2_ laser combined with a roller furnace is applied [29]. The CO_2_ laser, with a wavelength of 10.6 μm, a pulse duration of 10 μs and a maximum power of 124 W, operates in the Q-switch regime with a repetition rate of 12 kHz and a scan speed of 90 cm/s. A flat field lens with a focal length of 1 m is employed to focus the beam over the surface of the substrate, providing a working area of 120 × 120 mm^2^. The sample is gradually heated in a roller furnace at a speed of 1000 mm/h to 500 °C, a temperature below the transition temperature of the soda-lime glass but enough to avoid possible cracks during the process. When the channel reaches the central part of the furnace, the CO_2_ laser interacts with the surface of the soda-lime glass, reaching a temperature value at the top surface of the material above the transition temperature. This results in the melting of the material located in the upper part of the sample. The material in the channel melts and redistributes, leading to a structure with lower roughness. After the laser thermal treatment, the sample leaves the roller furnace in a cooling region. Figure 1 shows the experimental setup for the fabrication of the channel over soda-lime glass with a laser that will act as a master for the final channels in PDMS.

As we can see in Figure 1a, the laser is focused on the metal target and the particles ejected cause the ablation of the soda-lime glass. Using this method the channel with the desired geometry is achieved by applying several laser passes. Figure 1b shows the thermal treatment with a CO_2_ laser that leads to an improved final structure of the master channel after four laser passes. In this manner, channels with a 1.215 ± 0.010 mm depth are achieved, finding that this value decreases until 1.005 ± 0.010 mm after the thermal treatment.

#### 2.1.2. Master Replica Using Soft Lithography Technique 

**Figure 2:**
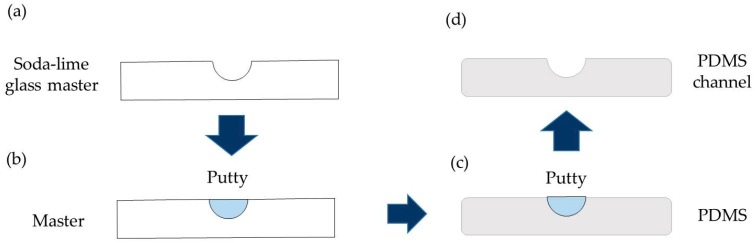
Scheme of the replication process. (**a**) Soda-lime glass master fabricated by laser techniques; (**b**) The putty is placed inside the channel to obtain the inverse structure of the master; (**c**) The putty mold is covered with PDMS and cured; (**d**) Finally, after peeling off the PDMS, the final channel is obtained.

Once the master is fabricated with laser techniques, we replicate it with PDMS using a well- known soft lithography method. We use an impression material, Aquasil Ultra Soft putty, in a ratio of 1:1 to obtain the inverse structure of the master. The putty is mixed and placed into the channel, practicing slight pressure. The mix is allowed to dry for one hour and then it is removed from the soda-lime channel, obtaining an accurate inverse mold of the channel. In order to recover the original structure, PDMS is employed. The putty mold is covered by PDMS in a ratio of 1:10 and it is degasified for one hour in a vacuum chamber at 120 mbar to avoid bubbles in the final structure. Then, the putty mold and the PDMS are inserted inside a static furnace at 40 °C for three hours to slowly cure the PDMS in order to prevent the formation of air bubbles that were not well degasified. During this procedure the putty is covered by a thin coat of epoxy, which does not alter the channel structure, to avoid the exchange of material between the putty and the PDMS throughout the curing process. Once the PDMS is cured, it is carefully peeled off from the putty and a replica of the soda-lime glass channel is obtained. In Figure 2, the soft lithography procedure to obtain the replica of the channel fabricated on soda-lime glass in PDMS is illustrated. This three-step process yields a precise copy of the master.

### 2.2. Endothelial Cell Seeding in PDMS Channels

**Figure 3:**
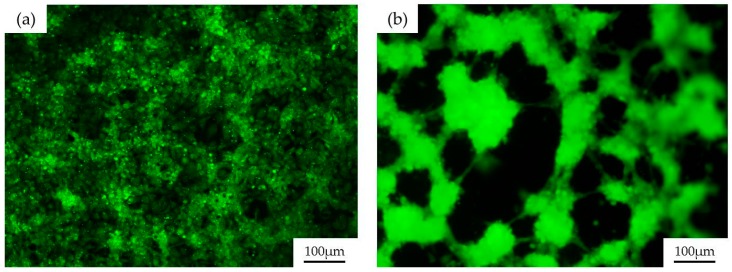
Representative fluorescence microscopy images of the human umbilical vein endothelial cells (HUVEC) growing over a PDMS channel after (**a**) the first and (**b**) the third use of the device; The channel was cleaned with ethanol. HUVECs were stained with green calcein AM.

As mentioned before, one of the problems with PDMS devices for biomedical assays is the degradation of the material when organic solvents are employed. For example, when cleaning the PDMS with ethanol to sterilize the channel and to reuse the device, the material degrades, leading to an abnormal cell culture in the next assays. This behavior is shown in Figure 3.

Figure 3 depicts the culture of human umbilical vein endothelial cells (HUVEC) over a PDMS channel fabricated as previously described, without sol-gel coating. Figure 3a depicts an image of the PDMS surface after a one-day endothelial cell culture. In these conditions, HUVECs grow forming a confluent monolayer of cells, with the characteristic “cobblestone” morphology of endothelial cells. Figure 3b shows cell growth over the same channel, when it is reused three times and washed with alcohol between usages for sterilization purposes. In these cases, HUVECs are far from forming a confluent monolayer. In contrast, they are placed over the PDMS, gathering in clusters, minimizing the surface that they share with the degraded PDMS. This situation happens with all of the organic solvents commonly employed in biomedicine and one easy solution to this problem, as was previously mentioned, is to coat the PDMS channels with sol-gel to confer the structure with the chemical robustness of the glass.

### 2.3. Sol-Gel Coatings

**Figure 4:**
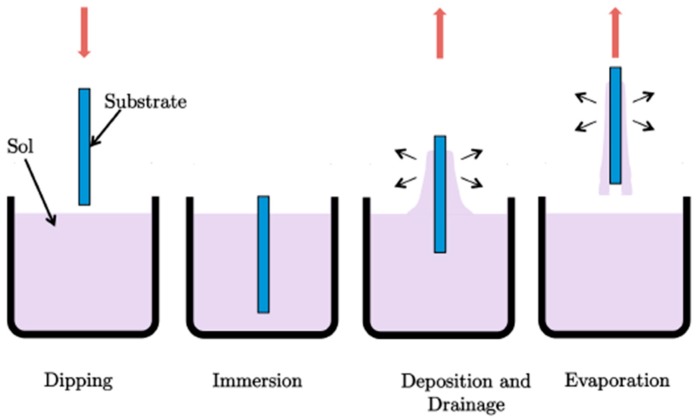
Deposition by the dip-coating technique: dipping and immersion of the substrate into the sol, formation of the layer by withdrawing the substrate and gelation of the layer by solvent evaporation.

Different silica and silica-titania sol-gel coatings are applied on PDMS channels by the sol-gel dip-coating technique using methyltriethoxysilane (MTES) and tetraethylorthosilane (TEOS) as silicon dioxide precursors and titanium isopropoxide (TISP) as a titanium dioxide precursor. In particular we use the 60MTES/40TEOS, 70MTES/30TISP and 80MTES/20TISP compositions. The preparation procedure is explained in detail in Section 4. In the dip-coating technique, the substrate to be coated is immersed in a liquid and subsequently withdrawn at a constant withdrawal speed. The process takes place under well-controlled temperature and atmospheric conditions. The film formation involves several steps but, nevertheless, the underlying physical and chemical processes are mostly overlapping. To obtain the final film, normally a thermal treatment is necessary. The densification temperature depends on the composition. The schematics of the dip-coating process are represented in Figure 4. Basically, the process starts with the immersion of the substrate in the coating bath. Next, the liquid film is entrained on the removal the substrate from the liquid, which then consolidates by drying and the accompanying chemical reactions. The consolidation step represents the sol-gel transition with the concomitant processes of draining, evaporation and hydrolysis.

**Figure 5:**
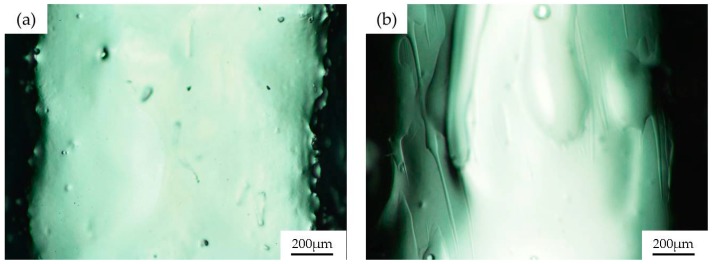
Microscopy images of PDMS channel: (**a**) uncoated and (**b**) with sol-gel coating.

After the fabrication process, the morphological analysis of the channels by means of optical microscopy reveals the presence of holes and irregular edges (see Figure 5a). The deposition of the sol-gel layers reduces the roughness of the surface without altering the shape of the channels, as shown in Figure 5b.

### 2.4. Biological Validation

Once the PDMS channels are coated with sol-gel to increase the chemical robustness of the structure, the endothelial cell behavior over the different coated surfaces is studied in order to verify its biocompatibility and to determine which sol-gel coating is the most suitable for endothelial cell growth.

**Figure 6:**
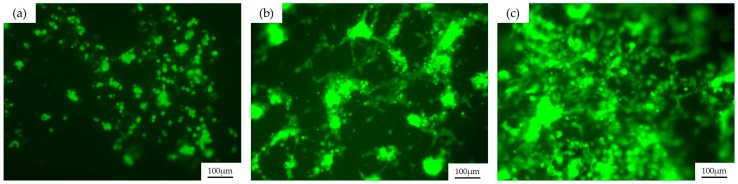
Representative fluorescence microscopy images of the different sol-gel–coated channels with HUVECs stained with calcein AM after a one-day culture. (**a**) PDMS channel with 60MTES/40TEOS coating; (**b**) with 70MTES/30TISP coating; and (**c**) with 80MTES/20TISP sol-gel coating.

For this purpose, we chose to culture HUVECs over the three different coated channels. PDMS channels are immersed in endothelial growth medium (EGM) for 30 min to enhance cell adhesion and then cells are seeded over the channels and are incubated for one day at standard conditions. Channels are washed with medium in order to remove the cells that are not adhered to the surface. The samples are observed under fluorescence microscopy and the results are shown in Figure 6.

As we can see in Figure 6, HUVECs stained with calcein—a viability indicator—grow over the three different coatings, so we can say that the sol-gel compositions presented here are all biocompatible. Nevertheless, significant differences in the growth and spread of the cells are observed between channels and they will be discussed in Section 3. We can appreciate in Figure 6 that even after washing the cell cultures with medium, there are still some remaining clusters of cells that attach to other cells instead of adhering to the surface of the material, generating an excess of fluorescent signal.

## 3. Discussion

In this work, PDMS devices that could mimic blood vessels for biomedical applications were manufactured. By using the laser direct writing technique for master fabrication over soda-lime glass, structures more than one millimeter in depth were achieved without losing the accuracy offered by laser technology. A thermal treatment using a CO_2_ laser was applied to the sample in order to obtain the final master with appropriated roughness, which is then replicated in PDMS by using conventional soft lithography methods. The final PDMS channels were coated with a glass-like layer using different sol-gel compositions (60MTES/40TEOS, 70MTES/30TISP and 80MTES/20TISP) to decrease the deterioration of the structure when bioassays are done using them several times, turning the devices into reusable ones. Due to the fact that these structures imitate blood vessel geometries, HUVECs were seeded over them in order to study the biocompatibility of the substrates. Endothelial cells were stained with calcein AM, a viability indicator that makes the cell fluorescent when it is alive. By means of fluorescence microscopy, we have observed that all sol-gel coatings allow the cells to live, but with important differences between them. Clearly, as we can see in Figure 6c, the 80MTES/20TISP sol-gel composition presented the most suitable environment for the growth and adherence of the HUVECs. A higher proliferation of cells compared with the other channels was observed and, moreover, they were more spread, almost forming a monolayer of cells. On the other hand, the 60MTES/40TEOS coating, shown in Figure 6a, presented the most hostile medium for HUVEC adhesion. Despite the fact that they were alive, cells did not spread over the surface and their morphology suggested a weak anchoring to the surface. The 70MTES/30TISP coating, depicted in Figure 6b, presented an intermediate situation, where we can see some of the endothelial cells attached and spread, but not as well as with the 80MTES/20TISP. In summary, despite showing that endothelial cells live over the three coatings, we can say that the 80MTES/20TISP sol-gel coating is the most appropriate composition when working with HUVECs due to the stretched form of the cells and their attempt to form a monolayer, which indicates that cells are attached well to the surface. 

This experiment was performed with endothelial cells because a blood vessel–like system was manufactured, but it is well known that the behavior of the cells over a substrate depends on the type of cultured cells used [30]. This work is a first approach in the study of the biocompatibility of different sol-gel coatings and these results could vary as a function of the kind of cell culture. In the future, this study is intended to be performed using different sorts of cells in order to establish the most suitable composition of sol-gel coating for cultures for each cell type . This could provide medical and biological researchers the chance to choose the best sol-gel coating for their biological assays. The possibility to fabricate a PDMS cover and to seal it to the channel immediately after the dip-coating process might also be a future step to obtain a sealed channel to perform flux experiments with a sol-gel coating that avoids the deterioration of the device.

## 4. Materials and Methods

For the master fabrication, a Rofin Nd:YVO_4_ laser (Plymouth, MI, USA) operating in Q-switch regime with a fundamental wavelength of 1064 nm and pulses of 20 ns was employed. For the thermal treatment of the sample an Easy Mark CO_2_ laser (Panduit, Tinley Park, IL, USA), with wavelength 10.6 μm and pulses of 10 μs, was used. The master was fabricated over a commercial soda-lime glass, provided by local suppliers. In the replica procedure, we employed Aquasil Ultra Soft putty impression material (Dentsply, Konstanz, Germany) in a ratio of 1:1 to obtain the inverse structure of the soda-lime channel, and then we recovered the initial structure with polydimethylsiloxane PDMS Sylgard 184 (Dow Corning, Midland, MI, USA) in a ratio of 1:10. A Pfeiffer vacuum chamber was employed to degasify the PDMS and a Nanetti furnace was used to cure it.

**Figure 7:**
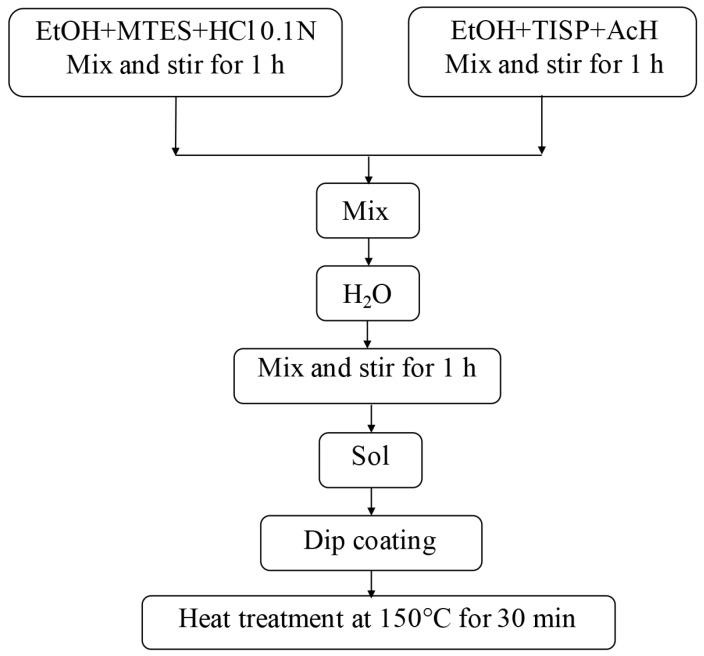
Flow chart of the preparation process of SiO_2_-TiO_2_ thin films.

Two MTES/TISP sols in the molar ratios 70:30 and 80:20 were prepared using MTES (CH_3_Si(OCH_2_CH_3_)_3_, 98%, ABCR GmbH & Co., Karlsruhe, Germany) and TISP (Ti[OCH(CH_3_)_2_]_4_, 97%, ABCR GmbH & Co., Karlsruhe, Germany) as silicon dioxide and titanium dioxide precursors, respectively, in acid environment. The preparation process was identical for both sols and was conducted in two steps. Initially, MTES was pre-hydrolyzed in the presence of HCl (0.1 N) with ethanol as solvent. On the other hand, TISP was dissolved in ethanol and complexed by adding glacial acetic acid (AcH). After stirring for one hour, the two parts were then mixed and distilled water was added drop by drop until hydrolysis was completed. The sol was synthesized at ambient atmosphere and room temperature. The molar ratios used for the obtaining of the sols with final concentration of 100 g/L are shown in Table 1 and Table 2. The synthesis route followed for the preparation of silica-titania layers is shown in Figure 7.

**Figure 8:**
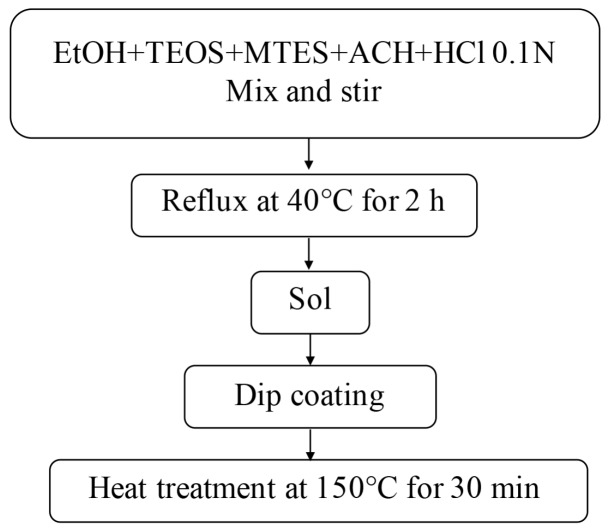
Flow chart of the preparation process SiO_2_ layers using MTES and TEOS.

A MTES/TEOS sol in the molar ratio 60:40 was prepared using TEOS and MTES as a mixed silica precursor. In this case, a two-step sol was prepared by adding water and acetic acid to the refluxed sol-gel process using TEOS (Si(OCH_2_CH_3_)_4_, 99%, ABCR GmbH & Co., Karlsruhe, Germany) and MTES (CH_3_Si(OCH_2_CH_3_)_3_, 98%, ABCR GmbH & Co., Karlsruhe, Germany) as silica precursors for the preparation of silica sol by hydrolysis and condensation reactions. First, TEOS and MTES were mixed with absolute ethanol and acidulated water (0.1 M AcH) was added drop by drop. Next, distilled water was incorporated to the solution. The sol was then refluxed in a water bath at 40 °C under continuous stirring for 2 h. All steps were carried out under ambient atmosphere. The molar composition of the sol with final concentration 180 g/L is listed in Table 3 and the flow chart followed for the preparation of Er3^+^-doped silica films is illustrated in Figure 8.

Films were deposited on the PDMS devices by dip-coating technique using a withdrawal rate of 6 cm/min. The deposited coatings were allowed to dry for several minutes before being removed for further thermal processing. After drying, the samples were heat treated at 150 °C for 2 h using a ramp rate of 5 °C/min in air atmosphere. With sol-gel process, coatings with high optical quality using low sintering temperature can be obtained, which is crucial in view of future industrial applications.

For the cell culture, we employed HUVEC that were obtained from umbilical cords donated after informed consent from the mothers. This protocol was approved by the Ethics Committee for Human Studies in Galicia (Spain) in accordance to the Declaration of Helsinki (1975). The HUVEC were isolated and cultured following the protocol described by Rodiño-Janeiro et al. [31]. Endothelial cells were cultured in complete endothelial growth medium (EGM-2; Lonza, Basle, Switzerland) supplemented with gentamicin sulfate/amphotericin B under standard cell culture conditions (37 °C temperature, more than 80% humidity and 5% CO_2_). HUVEC were stained with calcein AM (Invitrogen, Thermo Fischer Scientific, Waltman, MA, USA) for 4 min at 37 °C, which is a cell-permeant dye that is converted into green-fluorescent calcein in those cells that are alive. After dye loading, cells were washed twice in medium in order to remove the excess of calcein. HUVEC were seeded over the channels, previously sterilized in an autoclave (120 °C, 30 min), at a density of 10^6^ cells/mL and incubated for one day.

Samples inspection was performed using a Nikon MM-400 microscope (Tokyo, Japan). HUVEC cells were observed by means of fluorescence microscopy using an Axio Vert.A1 Zeiss microscope (Oberkochen, Germany).

## Figures and Tables

**Table 1:** Composition of the 70MTES/30TISP sol.

Composition	Molar Ratio
MTES/TISP	70/30
H_2_O/Alcoxides	1.5
Alkoxides/AcH	1

**Table 2:** Composition of the 80MTES/20TISP sol.

Composition	Molar Ratio
MTES/TISP	80/20
H_2_O/Alcoxides	1.5
Alkoxides/AcH	1

**Table 3:** Composition of the 60MTES/40TEOS sol.

Composition	Molar Ratio
MTES/TEOS	60/40
H_2_O/Alcoxides	1.8
Alkoxides/AcH	4
